# *PsnMYB30* Enhances Salt and Drought Stress Tolerance in Transgenic Tobacco

**DOI:** 10.3390/plants14172681

**Published:** 2025-08-27

**Authors:** Yuting Wang, Msangi Shamsia Ally, Ruiqi Wang, Wenjing Yao, Tingbo Jiang, Huanzhen Liu

**Affiliations:** 1 State Key Laboratory of Tree Genetics and Breeding, Northeast Forestry University, Hexing Road, Harbin 150040, China; wyt1513026188@163.com (Y.W.); msangishams@gmail.com (M.S.A.); 870831997@nefu.edu.cn (R.W.); yaowenjing@njfu.edu.cn (W.Y.); tbjiang@nefu.edu.cn (T.J.); 2 Bamboo Research Institute, Nanjing Forestry University, 159 Longpan Road, Nanjing 210037, China

**Keywords:** *Populus simonii × Populus nigra*, *PsnMYB30*, abiotic stress, ectopic expression, salt and osmotic tolerance

## Abstract

Drought and salinity are two major environmental factors that severely limit plant growth and development. MYB functions as a transcription factor that is crucial in how plants respond to stress from adverse conditions. In this study, we identified a gene encoding *Populus simonii × P. nigra* MYB (v-myb avian myeloblastosis viral oncogene homolog) transcription factor, whose transcription level was significantly induced under salt stress and osmotic stress. Subcellular localisation results showed *PsnMYB30* was located in the nucleus. Yeast one-hybrid assay indicated the gene exhibited transcriptional activation activity and it can precisely bind to the G-box elements. Under normal growth conditions, there were no significant differences in physiological and biochemical indicators between wild-type and transgenic tobacco. However, under salt and drought stress, transgenic tobacco overexpressing *PsnMYB30* exhibited superior root length and fresh weight compared to the wild-type (WT), with higher levels of SOD, POD, proline, and chlorophyll content, and significantly lower MDA and H_2_O_2_ content than the WT. These findings indicate that *PsnMYB30* significantly enhances the salt tolerance and drought resistance of transgenic tobacco. These results indicate that PsnMYB30 is a key target gene for studying salt-tolerant and drought-resistant plants in genetic breeding.

## 1. Introduction

Poplar (*Populus* L.) is characterised by rapid growth, widespread distribution, and dense foliage, making it an important tree species for ecological restoration and afforestation in arid and semi-arid saline-alkali regions [1]. However, its growth and yield are severely limited by factors such as drought, high salinity, and temperature [2]. In order to adapt to various adverse conditions, plants have evolved complex defence mechanisms that can activate or inhibit the expression of relevant resistance genes, thereby helping plants cope with stress [3,4]. Plants’ defensive responses to abiotic stress include signal transduction, gene expression regulation, ion homeostasis, reactive oxygen species scavenging, and other pathways [5]. Therefore, analysing the response mechanisms of plants to abiotic stress, exploring how poplar trees perceive and transmit stress signals, and how they regulate gene expression, can provide important information for breeding salt-tolerant and drought-resistant poplar trees [6].

Growing evidence suggests that numerous plant-specific transcription factor (TF) families mediate stress responses in plants [7,8]. Certain groups of transcription factors, like WRKY (WRKY Transcription Factor), NAC (No Apical Meristem, Arabidopsis Transcription Activation Factor, Cup-Shaped Cotyledon), bHLH (Basic Helix-Loop-Helix), and MYB (v-myb avian myeloblastosis viral oncogene homolog), play really important roles in how plants respond to various stresses, whether they come from living things or the environment [9,10]. In particular, the MYB family is a prominent central hub regulating stress-related gene expression in plants, which contains a growing population of R2R3-type MYB proteins [11]. The DNA-binding motif at the beginning of the MYB protein structure contains many repetitive tandem sequences, which is what makes it unique. [12]. Each repeat typically contains coding for 50–53 amino acids and three alpha helices, with the second and third helices forming a helix helix (HTH) structure [12]. The three tryptophan residues in the MYB domain are spaced at regular intervals of approximately 18 amino acids, which is important for maintaining the hydrophobic structure of HTH [13]. In addition, based on the number and position of MYB Rs repeat sequences in the structural domain, they can be divided into four different subfamilies: R2R3-MYB, MYB1-R, 4R-MYB, and R1R2R3-MYB [14]. These members function in cell differentiation, organ development, leaf shape, and responses to secondary metabolites and environmental stresses in plants [15,16]. Overexpression of MYB6 in poplar up-regulated the expression of flavonoid biosynthesis genes and significantly increased anthocyanin and proanthocyanidin accumulation [17]. For instance, excessive expression of MdMYB46 increases apple’s resistance to salt and osmotic stress by direct activation of stress response signalling pathway [18]. *OsMYB91* would be induced to express by salt stress, and transgenic plants overexpressing *OsMYB91* showed greater tolerance to salt stress. While various MYB family genes have been recognised across numerous plant species, a significant number of MYB members in poplar remain uncharacterised in terms of their functions [19].

In the analysis, it was found that the expression of *PsnMYB30* was significantly induced by salt stress, leading us to hypothesise that this gene may be involved in the salt stress response process of poplar. To verify this hypothesis, the *PsnMYB30* gene was cloned, an overexpression vector was constructed, and it was transferred into tobacco (*Nicotiana tabacum* L.) to identify its stress-resistant function. The results showed that *PsnMYB30* is located in the cell nucleus, possesses transcriptional activation activity, and can specifically bind to the G-box element. The salt tolerance and drought resistance of transgenic tobacco overexpressing *PsnMYB30* were tested under adverse conditions. Physiological and biochemical results indicated that the overexpression of *PsnMYB30* enhanced the salt tolerance and drought resistance of transgenic tobacco, confirming the biological function of *PsnMYB30* in the stress response of poplar.

## 2. Methods and Materials

### 2.1. Plant Materials

Tissue culture seedlings of *P. simonii × P. nigra* grown in the greenhouse of Northeast Forestry University for DNA and RNA isolation [20].

First, rinse the tobacco (*Nicotiana tabacum* L.) seeds with anhydrous ethanol for 15 s, then soak them in a 20% sodium hypochlorite solution for 8 to 10 min. In the second step, the washed seeds are cultured on MS medium, rinsed three times with sterile water, and allowed to dry before being cultured until germination. The seedlings are then transferred to MS culture bottles for continued cultivation for four weeks. Well-developed leaves are harvested and subjected to transgenic transformation using the *Agrobacterium*-mediated leaf disc method, with the process continuing.

### 2.2. Sequence Analysis of PsnMYB30

RNA extracted from the leaves of *P. simonii × P. nigra* was purified using Total RNA Purification kits provided by Gene-star (https://www.Gene-star.com/, accessed on 5 June 2025) (Beijing, China). That transcript sequence of *PsnMYB30* (Potri.008G166700) was retrieved from the PlantTFDB5.0 database. The cloning primers, *PsnMYB30F1* and *PsnMYB30R1*, were designed according to the characteristics of the *PsnMYB30* sequence (Supplementary Table S1).

Protein sequences similar to PsnMYB30 were retrieved from the NCBI database (http://blast.ncbi.nlm.nih.gov, accessed on 6 June 2025) using BLASTP from nine different plant species. In MEGA 11, the Neighbour-joining method and Maximum Likelihood (ML) were used to construct a phylogenetic tree (bootstrap = 1000). The structure of the PsnMYB30 protein was predicted using SWISSMODEL based on the example requirements [21].

### 2.3. Cis-Elements Analysis of Promoter Sequence

Extract DNA from *P. simonii × P. nigra* leaves and use the specific primers *PsnMYB30F2* and *PsnMYB30R2* to clone the promoter fragment (1498 bp) upstream of the ATG of the *PsnMYB30* gene (Supplementary Table S1). Through PlantCARE, cis-acting elements on the cloned promoter fragments were predicted, and cis-regulatory elements in the promoter sequence were identified and mapped using TBtools 2.147 and MEME Suite 5.3.0. [22].

### 2.4. Spatiotemporal Expression Analysis of PsnMYB30

Tissue culture seedlings of *P. simonii × P. nigra* were treated with 200 mM NaCl and 20% PEG-6000 for 0, 6, 9, 12, 24, and 48 h at one month of age, respectively. Collect leaves, stems, and roots to extract RNA. Use the actin gene as the internal reference gene for RT-qPCR and estimate the relative expression level of the gene using the 2^−ΔΔCt^ method (perform three biological replicates for each sample) [23]. Primers used for RT-qPCR can be found in Supplementary Table S1.

### 2.5. Subcellular Localisation of PsnMYB30

The pBI121-GFP vector was used to recombine the *PsnMYB30* CDS sequence without a stop codon. The fusion vector driven by CaMV 35S and 121-GFP empty vector were introduced into onion epidermal cells by particle bombardment. The fluorescence signals were examined using an LMS800 laser confocal microscope.

### 2.6. Yeast Hybrid Assay and Dual-Luciferase Reporter Assay

To determine the transcriptional activation activity of PsnMYB30, yeast hybridisation experiments were conducted. The coding sequence of PsnMYB30 was divided into six fragments, including MYB30a (0–120aa), MYB30b (121–287aa), MYB30c (121–246aa), MYB30d (205–287aa), MYB30e (205–242aa), and MYB30f (205–234aa). Recombinant plasmids pGBKT7-MYB30 (a–f) and pGBKT7-MYB30 were used as negative and positive controls, respectively, and were transformed into Y2H yeast cells. The yeast cells undergoing transformation were grown for about three to five days at 30 °C in SD/-Trp and SD/-Trp/-His/X-a-Gal medium. In addition, we used yeast one-hybrid experiments to verify whether PsnMYB30 can specifically bind to the G-box (TACGTG).

The PsnMYB30 protein’s ability to bind to the G-box was further validated using a dual luciferase reporter gene assay. The full-length coding sequence of PsnMYB30 was inserted into the pGreenII-62-SK vector. To create the pGreenII 0800-LUC vector, a triplicate repeat sequence of the G-box was constructed. The recombinant vector was transformed into the GV3101 strain containing the pSoup helper plasmid. In addition to using pGreenII 62-SK-PsnMYB30/pGreenII 0800-LUC, pGreenII 62-SK/pGreenII 0800-LUC and pGreenII 62-SK/pGreenII 0800-G-box were used as negative controls. We also injected pGreenII 62-SK-PsnMYB30 and pGreenII 0800-G-box into the leaves of three-week-old tobacco seedlings. After 40 h, D-fluorescein was applied to the leaves of the transgenic tobacco plants, and the resulting fluorescence was recorded using an in vivo plant imaging system (Tanon 5200 Multi).

### 2.7. Acquisition of Transgenic Tobacco

The pBI121 vector was modified by injecting the *PsnMYB30* CDS at the XbaI and SpeI restriction sites (the carrier was purchased from Changsha Aibivei Biotechnology Co., Ltd.; see Supplementary Figure S1 for plasmid diagram Changsha, China). The chosen positive strains were cultivated in LB liquid medium that had 50 mg/L of rifampicin and 50 mg/L of kanamycin until the optical density at 600 nm (OD600) hit between 0.5 and 0.8. After that, the resulting recombinant vector was injected into the *A. tumefaciens* strain GV3101. One-month-old tobacco seedling leaves were cut into rectangular shapes and soaked in the bacterial solution for a time length of 8 to 10 min. The leaves were subsequently plated on the MS medium containing 50 mg/L kanamycin and 200 mg/L ceftriaxone sodium. To produce resistant seedlings, the resistant shoots were cultivated in MS with ceftriaxone and kanamycin. By using specific primers, DNA was taken out from the leaves of these resistant seedlings for molecular identification in order to verify the positive transgenic lines (Supplementary Table S1). DNA of wild-type (WT) samples and water were used as negative controls, while the recombinant plasmids were served as positive control (Supplementary Figure S2). T1 seeds of three randomly selected positive tobacco lines were harvested and propagated to obtain T2 and T3 seeds.

### 2.8. Histochemical Staining and Physiological Measurement

At the four-leaf stage, the T3 transgenic tobacco seedlings were put into rooting media that contained 20% PEG-6000 and 200 mM NaCl, respectively. Three biological replicates were used to measure fresh weight and root length of the tissue culture seedlings after 15 days. At one month old, the T3 transgenic seedlings were subjected to 200 mM NaCl and 20% PEG-6000 for 6 h, respectively. Subsequently, the leaves were collected and stained separately with nitroblue tetrazolium chloride (NBT) and 3,3′-diaminobenzidine (DAB). The degree of stress on the leaves was determined based on the stained area and intensity. Seven days later, the collected leaves were used for physiological measurements. The physiological indicator detection kit was provided by Shanghai Biotechnology Co., Ltd. (https://www.shbio.com/, accessed on 10 June 2025), and the specific experimental methods were carried out according to the kit instructions. The company is located in Shanghai, China.

## 3. Results

### 3.1. Expression Pattern of MYB Family Genes in Poplar Roots Under Salt Stress

**Figure 1:**
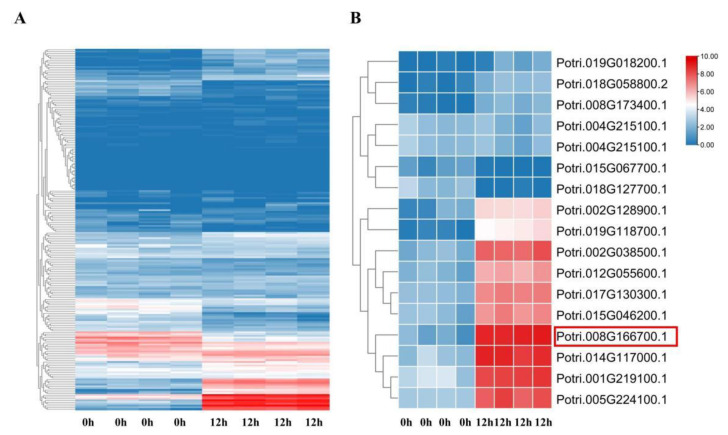
Heatmaps of MYB family gene in *Populus simonii × P. nigra* roots under salt stress. (**A**) Expression patterns of 207 MYBs. (**B**) Expression patterns of 17 DEGs; the colours blue and red are used to indicate expression levels, with blue representing low expression and red signifying high expression. The coloured scales demonstrate variations in transcript levels due to changes in ploidy. The red boxes indicate candidate genes.

We extracted FPKM information of 207 MYBs from RNA-Seq data of *P. simonii × P. nigra* roots treated with NaCl for 12 h in order to examine the expression pattern of MYB family genes in poplar under salt stress (Figure 1A) [24]. Differentially expressed genes (DEGs) were identified when the *p*-value was less than 0.05 and the absolute value of log2 (FC) was more than 1 (See Supplementary Table S2 and Figure 1B). Out of 17 DEGs that were evaluated, *PsnMYB30* was chosen as potential gene for functional validation since it was considerably upregulated in the root during salt stress.

### 3.2. PsnMYB30 Sequence Analysis

**Figure 2:**
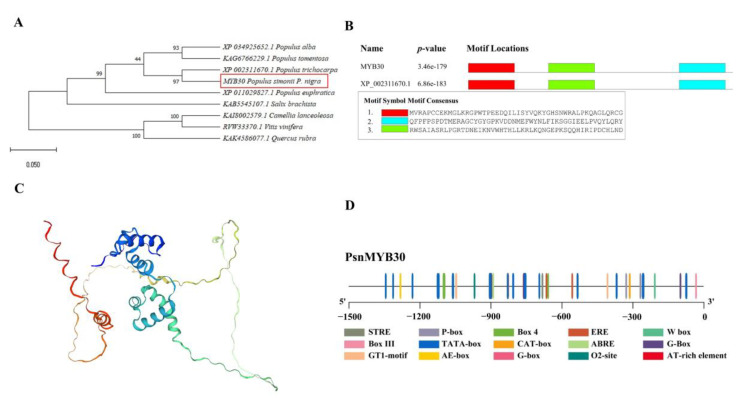
Sequence analysis of PsnMYB30. (**A**) Evolutionary analysis of *PsnMYB30* and nine other homologous genes. (**B**) Conserved structural domain of *PsnMYB30*. (**C**) Protein structure prediction of *PsnMYB30*. (**D**) Cis-element analysis of *PsnMYB30* upstream promoter.

The CDS length of *PsnMYB30* is 861 bp, which corresponds to 287 amino acids (aa). A highly conserved MYB domain can be found in the N-terminus contains. Eight proteins were found that are extremely similar to *PsnMYB30* from *Populus trichocarpa* (XP_002311670.1), *P. alba* (XP_034925652.1), *P. euphratica* (XP_011029827.1), *P. tomentosa* (KAG6766229.1), *Salix brachista* (KAB5545107.1), *Quercus rubra* (KAK4586077.1), *Camellia lanceoleosa* (KAI8002579.1), and *Vitis vinifera* (RVW33370.1) with protein sequence similarities of 97.21%, 95.12, 93.73%, 94.43%, 88.24%, 63.44%, 59.57%, and 55.91%, respectively (Figure 2A). According to NCBI blast, *PsnMYB30* shares three highly conserved structural domains with the MYB from *P. trichocarpa* (XP_002311670.1) (Figure 2B). The protein includes 29.27% alpha helix, 2.44% extended chains, 4.18% beta bends, and 64.11% random coils (Figure 2C). The 1498 bp upstream promoter sequence of *PsnMYB30* contains typical regulatory elements such as W-box, CAT-box, and TATA-box, light-responsive elements like G-box, GT1 motif, Box 4, and AE-box, and phytohormone response elements including ABRE and P-box. In particular, the stress response element STRE is also included in the promoter sequence of *PsnMYB30* (Figure 2D).

### 3.3. Spatiotemporal Expression Pattern of PsnMYB30 in Poplar

**Figure 3:**
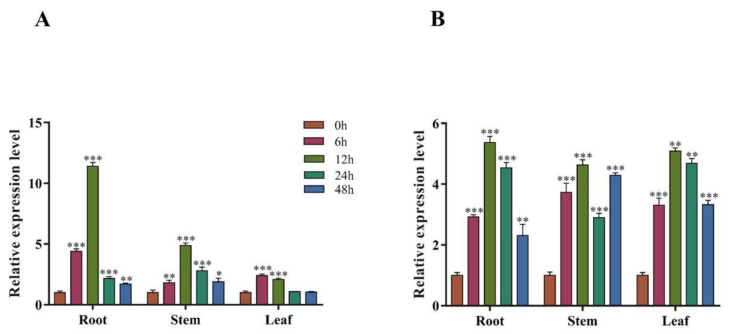
Spatiotemporal expression pattern of *PsnMYB30* in *Populus simonii × P. nigra*. (**A**) Expression pattern of *PsnMYB30* under 200 mM NaCl stress. (**B**) Expression pattern of *PsnMYB30* under 20% PEG-6000 stress. Note: * *p* < 0.05, ** *p* < 0.01, *** *p* < 0.001.

The spatiotemporal expression results of RT qPCR showed that under two stress conditions, the expression pattern of this gene was consistent, mainly enhanced in roots, with little change in expression in leaves and stems. Additionally, the most significant level of expression was detected in the roots at the 12 h mark when subjected to both types of stress. (Figure 3 and Supplementary Table S3).

### 3.4. Subcellular Localisation of PsnMYB30 Protein

**Figure 4:**
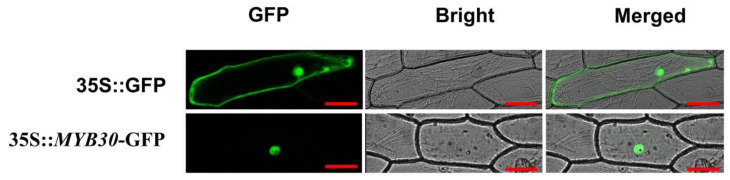
Subcellular location of *PsnMYB30* protein. The image includes a dark-field image (GFP), a bright-field image (Bright), and a superimposed image (Merged). The zoom bar is 20 µm.

Using particle bombardment, the control vector pBI121-GFP and the recombinant 35S:*PsnMYB30*-GFP were temporarily converted into onion epidermal cells. As seen in Figure 4, the fluorescence sign of the recombinant vector was located in the nucleus while it was in the whole cell for the control vector, indicating *PsnMYB30* was expressed in the nucleus.

### 3.5. Transcriptional Activation Activity of PsnMYB30

**Figure 5:**
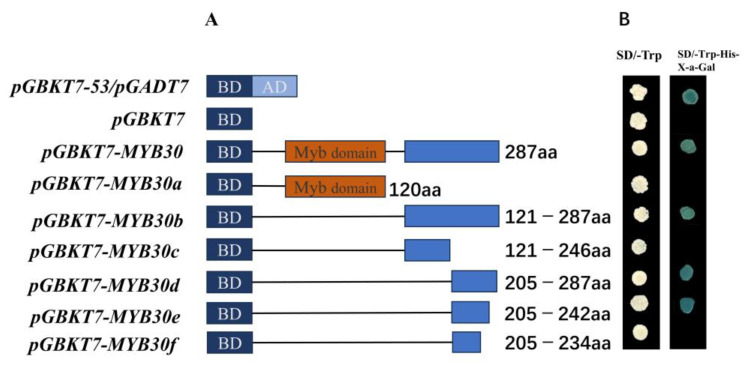
Yeast one-hybrid assay. (**A**) Schematic representation of *PsnMYB30* and its individual segments. The orange and blue colours represent the conserved domain and the non-conserved parts of *PsnMYB30*, respectively. (**B**) Segmented yeast colony colour response.

Positive and negative control plasmids and the full-length sequence of *PsnMYB30* and its six segmented sequences were transformed into Y2H receptor cells (Figure 5A). The only plasmids that grew normally on SD/-Trp/-His/X-a-Gal medium were the positive control plasmid (pGBKT7), pGBKT7-MYB30, pGBKT7-MYB30b, pGBKT7-MYB30d, and pGBKT7-MYB30e, and produced a colour reaction. And all the strains grew well on DDO medium (Figure 5B). This indicates that the shortest transcriptional activation region of PsnMYB30 is located at 205–242aa.

### 3.6. Specific Binding of PsnMYB30 to G-Box

**Figure 6:**
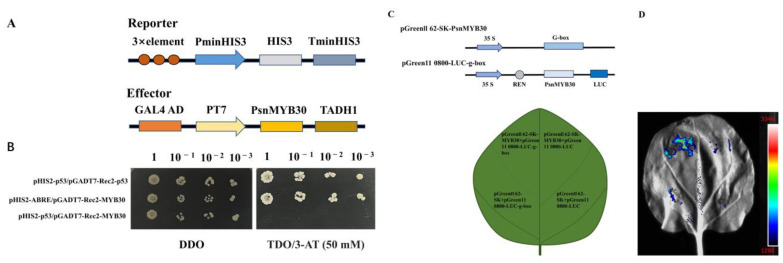
*PsnMYB30* specific binding to G-box. (**A**) Reporting and effector carrier schematics. (**B**) Yeast cells that were transformed were examined on DDO and TDO/3-AT (50 mM) media at various diversities. (**C**) Schematic diagram of recombinant vectors. (**D**) Transient expression shows that *PnsMYB30* binds specifically to G-box element.

The pHIS2 and pGADT7-Rec2 vectors were utilised to recombine with three tandemly repeated G-box elements and recombinant *PsnMYB30*, respectively (Figure 6A). In strain Y187, the pHIS2-G-box/pGADT7-Rec2-MYB30, negative control, and positive control were co-transfected, respectively. As illustrated in Figure 6B, all transformed cells displayed normal growth on DDO medium. Nevertheless, on TDO/3-AT (50 mM) media, only the positive control and pHIS2-G-box/pGADT7-Rec2-MYB30 displayed typical growth. These results suggest that *PsnMYB30* can specifically bind to G-box components.

Additionally, transient expression studies demonstrated that tobacco leaves fluorescently expressed pGreenII 62-SK-*PnsMYB30*/pGreenII 0800-G-box (Figure 6D). And the absenteeism of fluorescence in the leaves co-transformed with pGreenII 62-SK/pGreenII 0800-G-box, pGreenII 62-SK-*PnsMYB30*/pGreenII 0800-LUC, and pGreenII 62-SK/pGreenII 0800-LUC also confirmed that *PnsMYB30* binds precisely to G-box.

### 3.7. Morphological Features of Transgenic Tobacco Under Stress Conditions

Seedlings from tissue culture that exhibited comparable growth conditions were utilised to examine their morphological characteristics when subjected to stress. No notable disparity in growth indices was observed between the wild type and the transgenic plants when subjected to normal conditions. Under 200 mM NaCl and 20% PEG-6000, the transgenic lines showed better growth status than wild-type (WT). The transgenic lines demonstrated notable benefits in root growth and fresh weight compared to WT when exposed to stress conditions involving NaCl and PEG-6000 (Figure S3 and Supplementary Table S4).

### 3.8. Histochemical Staining of Transgenic Tobacco

**Figure 7:**
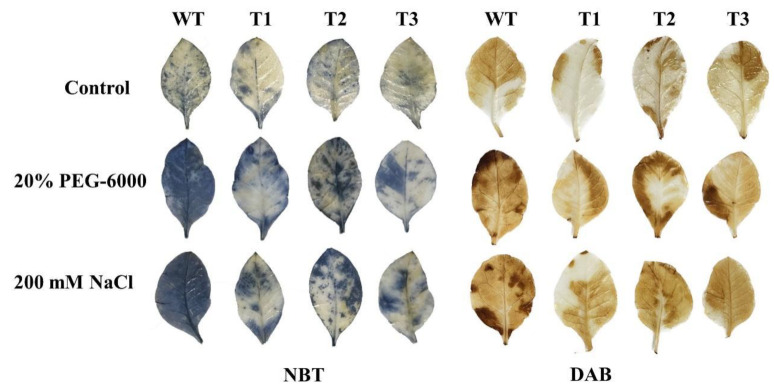
Nitroblue tetrazolium (NBT) and 3, 3′-diaminobenzidine (DAB) staining.

Transgenic tobacco was subjected to NBT and DAB staining in order to assess the buildup of ROS in the leaves under stress. Under control conditions, no significant differences in staining intensity were observed between WT and transgenic tobacco. Nevertheless, the staining intensity and area for WT were noticeably greater than those of the transgenic tobacco when subjected to both salt and PEG stresses (Figure 7).

### 3.9. Physiological Changes in Transgenic Tobacco Under Stress Conditions

**Figure 8:**
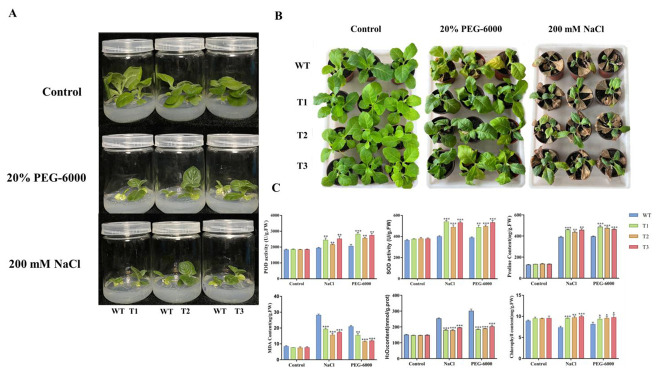
Stress-induced physiological changes in WT and transgenic tobacco plants (**A**,**B**). (**C**) Physiological indicators including the activity of SOD and POD, and the content of H_2_O_2_, MDA, proline, and chlorophyll. The error bars represent the mean ± standard deviation of at least three biological repeats (*t*-test, * *p* < 0.05, ** *p* < 0.01, *** *p* < 0.001).

We investigated into how transgenic tobacco changes under stress, focusing on things like SOD activity, hydrogen peroxide (H_2_O_2_) levels, chlorophyll content, POD activities, and the levels of MDA and proline (Figure 8 and Supplementary Table S5). The results reflect a notable SOD and POD activity in transgenic tobacco compared to WT under stress from salt. In transgenic tobacco, the levels of proline, chlorophyll, and hydrogen peroxide (H_2_O_2_) were notably enlarged in comparison to wild type (WT) upon exposure to salt stress. Conversely, transgenic tobacco exhibited reduced MDA in comparison to WT under salt and PEG stress conditions. The aforementioned results suggest that under both salt and PEG stresses, ROS accumulation in WT was greater than that in transgenic tobacco. The increased expression of *PsnMYB30* decreases ROS accumulation, increases proline accumulation, and protects cell membrane integrity in transgenic tobacco (Figure 8).

## 4. Discussion

Plant growth, development, and yield are affected by conditions such as high salinity, drought, and heat stress [25]. Significant climate change has an irreversible impact on all physiological and biochemical processes in plants [26]. There is mounting proof indicating that those multiple kinds of TFs mediate salt and drought stress responses in plants [27,28]. Among them, MYB TFs are significant in plant signalling, plant growth and development, and biotic and abiotic stress responses [29,30]. In poplar, a total of 207 R2R3-MYB genes have been identified and found to be unevenly distributed across 19 chromosomes [31]. However, only a small number of these MYB genes have been functionally characterised, indicating the need for further functional exploration of other poplar MYB genes [32].

This work found *PsnMYB30*, a highly salt-induced MYB gene in poplar roots, and examined its possible involvement in the response to drought and high salt stress. *PnsMYB30* was found to be localised in the nucleus with transcriptional activation activity locating 205–242aa. Additionally, it was demonstrated that *PnsMYB30* specifically binds to G-box element. ROS accumulation is one of the typical biochemical changes when plants are faced with adverse environmental stresses. Plants have evolved ROS scavenge ability to prevent cell damage from excessive ROS under stress conditions [33]. Typically, SOD, APX, CAT, and POD contribute significantly to the removal of excess ROS in plants as crucial enzymes in the antioxidant process [34]. The present study showed that the transgenic tobacco seedlings’ resilience to salt and drought was enhanced by the notable increase in SOD and POD activities. MDA is a common physiological indicator representing the degree of membrane lipid peroxidation in plants [35,36,37]. In this study, under stress circumstances, transgenic tobacco’s elevated *PnsMYBA30* expression resulted in lower MDA levels than wild type (WT). Proline is essential for maintaining the integrity of cellular membranes and is important for regulating osmotic balance. When exposed to salt and drought stressors, transgenic tobacco plants had a noticeably higher proline level than wild-type (WT) plants. Chlorophyll serves as the photosynthetic pigment, and a high concentration of it enhances plant growth in traumatic environments. According to the study’s data, transgenic plants exposed to PEG and salt stressors showed a considerable increase in proline accumulation and chlorophyll content. All the results indicate overexpression of *PsnMYB30* potentially enhances osmotic balance, thereby enhancing salt tolerance in transgenic tobacco.

Various studies have examined the molecular mechanisms by which transcription factors (TFs) specifically interact with cis-elements located in the promoters of downstream target genes, thereby regulating their expression [38,39]. For instance, *CsbZIP50* directly interacts with the G-box/ABRE cis-acting elements found in the promoter of *CsRD29* to increase drought resistance by affecting the expression of genes that react to drought. Similarly, *bHLH106* enhances salt tolerance of transgenic Arabidopsis through binding to G-box of multiple downstream genes [40,41]. In the study, it has been confirmed that *PsnMYB30* can specifically bind to G-box elements. However, it is unclear which downstream target genes can be regulated by *PsnMYB30* and whether *PsnMYB30* regulates the expression of these downstream genes by binding to G-Box in their promoters. These issues need to be further verified in future studies.

## 5. Conclusions

In this experiment, we observed the expression levels of MYB family members within the roots of *P. simonii × P. nigra* subjected to salt stress, discovering a notably salt-responsive MYB gene, *PsnMYB30*. This gene, PsnMYB30, was found to be located in the nucleus, with its region responsible for transcriptional activation identified between amino acids 205 and 242. *PnsMYB30* specifically binds to G-box (TACGTG) element. Furthermore, the transgenic tobacco plants overexpressing *PsnMYB30* displayed reduced levels of ROS accumulation, along with increased proline accumulation and cell membrane integrity when subjected to salt and drought stresses. Consequently, these modifications increased the stress resistance of genetically modified plants. Functional characterisation of *PsnMYB30* has contributed to the understanding of the role of MYB family members in stress response in poplar.

## Data Availability

The original contributions presented in this study are included in the article/Supplementary Material. Further inquiries can be directed to the corresponding author.

## Supplementary Materials

The following supporting information can be downloaded at: https://www.mdpi.com/article/10.3390/plants14172681/s1, Figure S1: pBI121-GFP Vector Map; Figure S2: Verification of transgenic tobacco. (A) DNA level test results. (B) Growth of transgenic tobacco in screening medium, transgenic tobacco on the left, WT on the right; Figure S3: Morphological characteristics of transgenic tobacco plants under stress environments (A,B) Comparison of phenotype, fresh weight and root length between transgenic tobacco and WT in tissue culture seedlings under stress environments; Table S1: Experiments involving all primers; Table S2: Transcriptome data of 207 MYB family members; Table S3: Data on the spatio-temporal expression pattern of PsnMYB30 in poplar trees; Table S4: Data on fresh weight and roots length of transgenic tobacco under stress conditions; Table S5: Measurement data of physiological indices of transgenic tobacco under stress conditions.
