# Supplementary figures and images for: PsnMYB30 Enhances Salt and Drought Stress Tolerance in Transgenic Tobacco
Source: Plants (Basel). 2025 Aug 27;14(17):2681. doi: 10.3390/plants14172681 (PMC12430057; doi:10.3390/plants14172681)

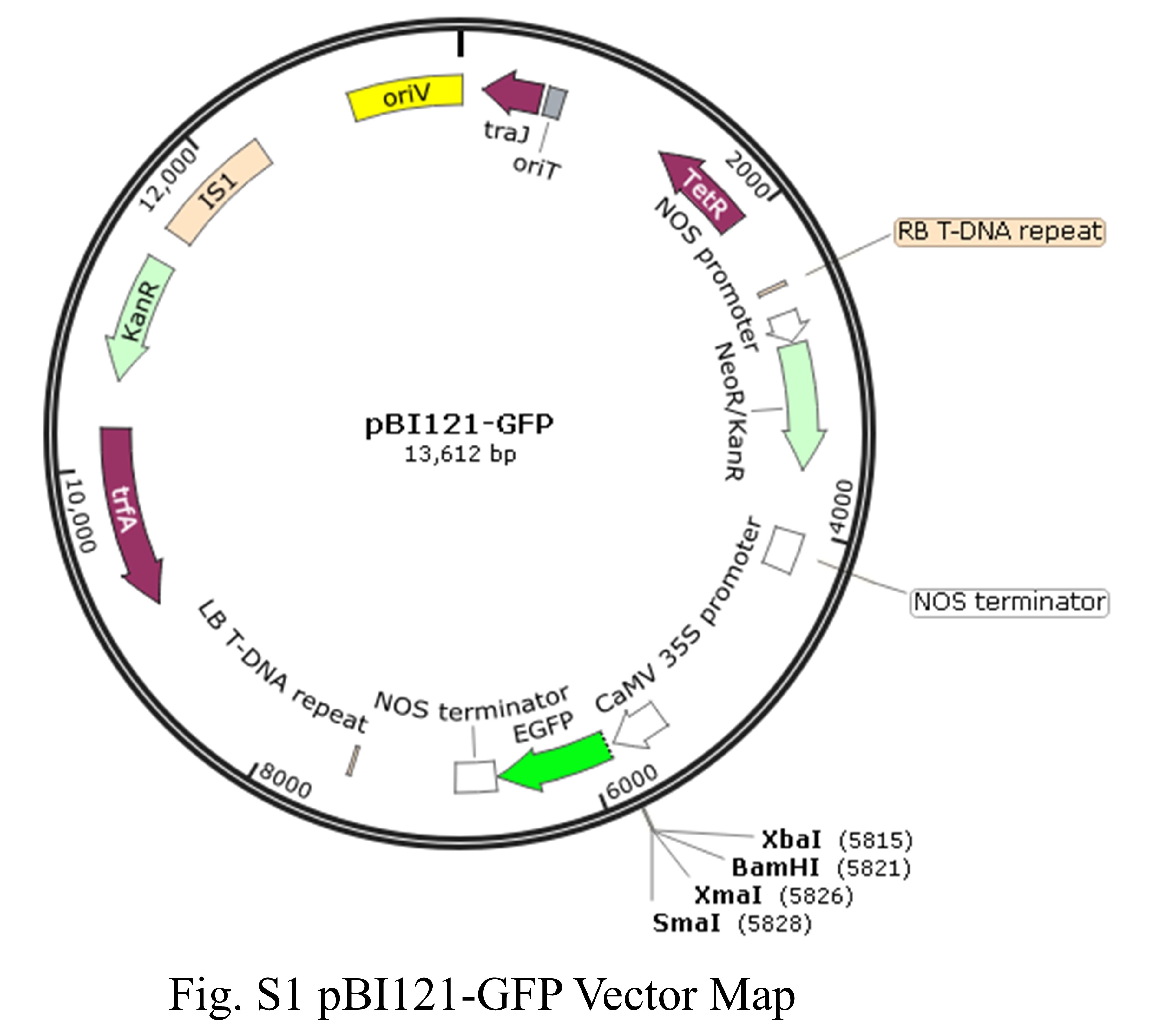

Supplement: Supplementary file 1 [file plants-14-02681-s001.zip › Fig.S1.jpg]

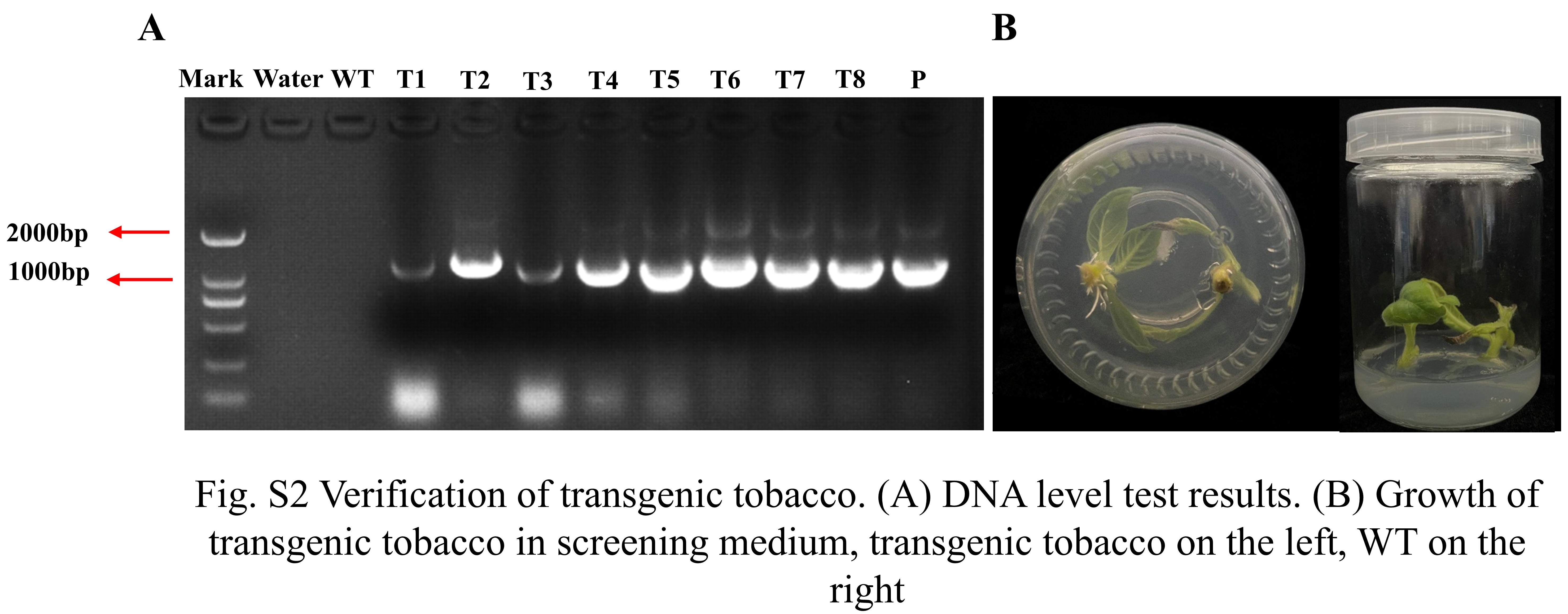

Supplement: Supplementary file 1 [file plants-14-02681-s001.zip › Fig.S2.jpg]

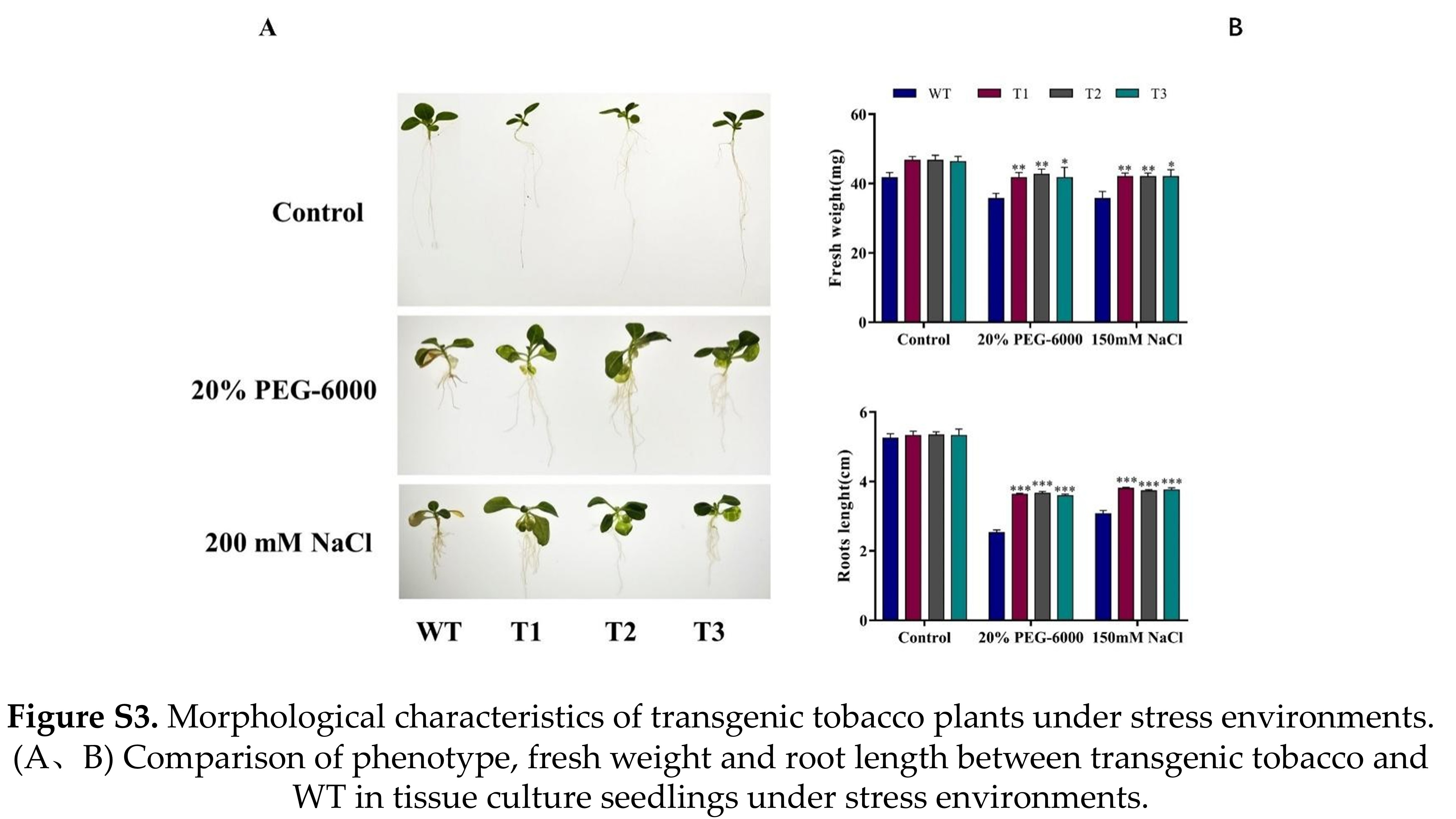

Supplement: Supplementary file 1 [file plants-14-02681-s001.zip › Figure S3.jpg]
